# Clinical characteristics, SARS-CoV-2 variants, and outcomes of adults hospitalized due to COVID-19 in Latin American countries

**DOI:** 10.1016/j.clinsp.2025.100648

**Published:** 2025-04-23

**Authors:** Constantino López-Macías, Eduardo López-Medina, Maysa Bonfleur Alves, Aline da Rocha Matos, Juan V. Hernández-Villena, Zuleika Aponte-Torres, Laura E. Sarabia, Paula Manrique-Ramirez, Luis F. Tejado-Gallegos, Larisa Ramirez Gutierrez, Wilhelmine Meeraus, Bárbara Emoingt Furtado

**Affiliations:** a Unidad de Investigación Médica en Inmunoquímica, UMAE Hospital de Especialidades, Centro Médico Nacional Siglo XXI, Instituto Mexicano del Seguro Social (IMSS), Ciudad de México, Mexico; b Centro de Estudios en Infectología Pediátrica CEIP, Departamento de Pediatría, Universidad del Valle, Clínica Imbanaco, Grupo Quironsalud, Colombia; c Instituto D'Or de Pesquisa e Ensino – IDOR, Rio de Janeiro, RJ, Brazil; d Fundação Oswaldo Cruz – Fiocruz, Instituto Oswaldo Cruz, Laboratório de Vírus Respiratórios, Exantemáticas, Enterovírus e Emergências Virais, Rio de Janeiro, RJ, Brazil; e P95 Epidemiology & Pharmacovigilance, Leuven, Belgium; f Vaccines and Immune Therapies, BioPharmaceuticals Medical, AstraZeneca, Ciudad de México, Mexico; g Vaccines and Immune Therapies, BioPharmaceuticals Medical, AstraZeneca, Gaithersburg, USA; h Vaccines and Immune Therapies, BioPharmaceuticals Medical, AstraZeneca, Cambridge, UK

**Keywords:** COVID-19 vaccines, Hospitalization, Latin America, SARS-CoV-2 variants, Comorbidity

## Abstract

**Objectives:**

COVID-19 vaccines prevented severe disease outcomes worldwide. As part of a vaccine effectiveness study, the authors summarized demographic and health profiles, vaccination data, SARS-CoV-2 variants, and disease outcomes from patients hospitalized due to COVID-19-like symptoms in Latin America between February and December 2022.

**Methods:**

LIVE was an observational-prospective study with a test-negative case-control design (NCT05282017). Adults admitted with COVID-19-like symptoms were recruited across five Latin American countries. Patients were tested for SARS-CoV-2, and variants were identified through sequencing. Data were collected from medical records and interviews.

**Results:**

Participants recruited (536 cases, 250 controls) had a mean age of 60.5 years. COVID-19 cases (63.7, SD = 19.1) were slightly older than controls (53.8, SD = 20.4). Disorders of the cardiovascular system were the most prevalent comorbidities, and most participants (73.0 %) reported at least one comorbidity. COVID-19 cases mostly presented with moderate (51.1 %) disease. Overall, 50.6 % of participants were unvaccinated against COVID-19, with a higher percentage in cases (56.3 %) than in controls (38.2 %). Viral genomic analysis identified the most prevalent SARS-CoV-2 lineages, BQ.1.1 (11.9 %) among Omicron BA.5 and XBB.1.15 (2.5 %) among recombinant variants. The study was underpowered to estimate vaccine effectiveness due to the low number of COVID-19 hospitalization cases.

**Conclusion:**

The present study revealed key demographic and clinical characteristics of patients hospitalized due to COVID-19. Notably, an older age among confirmed cases, a substantial proportion of unvaccinated individuals, and the decrease in hospitalizations emphasize the complexity of the COVID-19 landscape in Latin America and the need for continued research to inform public health strategies.

## Highlights

• During the Omicron wave, hospitalized patients with COVID-19 were mainly older adults.
• Disorders of the cardiovascular system were the most prevalent comorbidities.
• More COVID-19 cases were unvaccinated individuals compared to the controls.
• Most COVID-19 cases were moderate, but in-hospital deaths occurred in ∼20 % of cases.

## Introduction

COVID-19 vaccines were licensed as of December 2020 in Latin America, but the shortage of vaccines worldwide hindered the pace of vaccination.^1^^,^^2^ High-risk populations, mainly Healthcare Workers (HCWs) and elderly people, were prioritized during the initial immunization phases, and roll-out expanded to the general population as vaccines became accessible.^1^^,^^3^ The most frequently administered COVID-19 vaccines in Latin America were the vaccines developed by AstraZeneca (ChAdOx1), Pfizer-BioNTech (BNT162b2), Sinovac (CoronaVac), Sinopharm (BBIBP-CorV), Janssen (Ad26.COV2.S), Moderna (mRNA-1273), and Gamaleya (Sputnik V) .^4^

COVID-19 Vaccine Effectiveness (VE) against infection declined over time due to waning immunity and the emergence of SARS-CoV-2 variants.^5^ However, vaccines remain effective against severe forms of COVID-19.^5^, ^6^, ^7^, ^8^ Notable variants like Alpha, Beta, Delta, and Omicron have exhibited distinct traits that potentially influence transmissibility, disease severity, vaccine efficacy, and diagnostic precision. In Latin America, the first waves of COVID-19 were driven by co-circulating variants including Alpha, Beta, Gamma, Lambda, and Mu.^1^^,^^9^ Delta was the dominant variant from mid-to late 2021 and was promptly displaced by Omicron in January 2022, which caused the highest number of daily COVID-19 cases reported throughout the pandemic.^10^^,^^11^ To protect the general population, health organizations recommended booster doses.^12^

Compared to European countries and the USA, there is limited real-world evidence of the health profiles of COVID-19 patients and the effectiveness of COVID-19 vaccines administered to the Latin American population against SARS-CoV-2 variants. This information is essential for national and regional governments to adjust the National Immunization Programs and continue to protect the population against severe disease. Latin American Vaccine Effectiveness (LIVE) was an observational, prospective study (NCT05282017) that aimed to estimate the VE of COVID-19 vaccines against hospitalization due to circulating SARS-CoV-2 variants. Despite including eight sites across Brazil, Colombia, Costa Rica, Mexico, and Panama, recruitment goals were not met, and the study was underpowered to assess VE due to a low number of COVID-19 hospitalizations during this pandemic stage.

Here, the authors describe the COVID-19 patients recruited into the LIVE study, including demographic and vaccination data, health profiles, SARS-CoV-2 variants, and disease outcomes to show the types of patients still being hospitalized for COVID-19, for informing public health strategies.

## Materials and methods

### Study design

The authors conducted an observational, prospective study, with a test-negative case-control design (ClinicalTrials.gov Identifier: NCT05282017), based on the active surveillance of hospitalized COVID-19-like cases undergoing RT-PCR or Rapid Antigen Testing (RAT) for SARS-CoV-2 in Brazil, Colombia, Costa Rica, Mexico, and Panama. As the authors aimed to assess VE of COVID-19 vaccines, mainly ChAdOx1, the participating countries were selected if they had at least a 30.0 % contribution of ChAdOx1 vaccine in their National Immunization Programs and a 40.0 % vaccination coverage of target populations at the onset of this study.

### Study population and settings

**Fig. 1:**
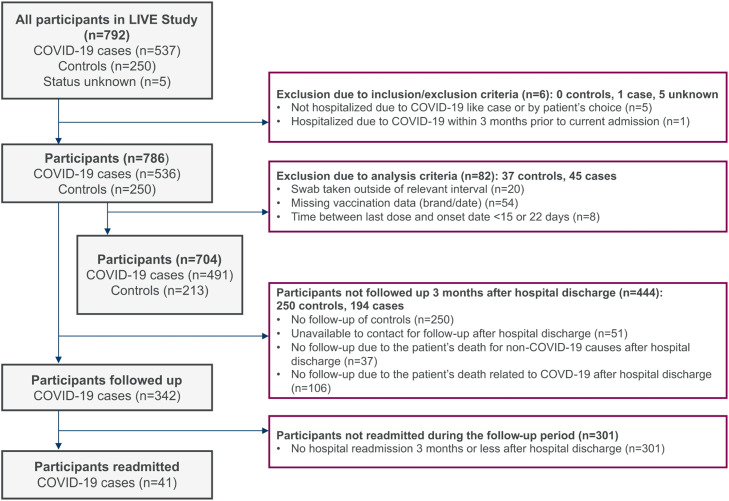
LIVE study attrition diagram.

All hospitalized patients admitted with COVID-19-like symptoms at the eight participating settings from February to December 2022, aged ≥18 years old, eligible for vaccination with ChAdOx1 or any other COVID-19 vaccines provided as per national/regional immunization recommendations, and willing to sign an Informed Consent Form (ICF) were eligible for this study (Supplementary Section 1). Each participant signed an ICF approved by the Institutional Review Board of each participating setting before undergoing any procedure related to this study (Supplementary Section 8). Patients who could not be swabbed for testing or were hospitalized due to COVID-19 three months prior to current admission were excluded (Fig. 1). The follow-up of the participants positive for SARS-CoV-2 began at admission and ended 3 months after discharge or at death (last patient last visit: April 2023) to record disease outcomes including readmission and death. Controls were not followed up.

The initial sample size considered was 2220 SARS-CoV-2 positive cases across five countries to detect a minimum VE of 70.0 % with sufficient precision (95 % Confidence Interval width of ≤ 50.0 %), as well as an overall strain-specific adjusted VE, where the prevalence of the circulating strain was at least 10.0 % overall and 40.0 % or above in a specific country (Supplementary Section 2).

### Data sources

Data were collected at the study settings using standardized questionnaires (Supplementary Section 3) during swabbing (with the patient or a relative in case of patient incapacity) and by consulting medical records from the participating sites, vaccination registries, vaccination cards, and laboratory test results. In addition, COVID-19 cases were followed up using face-to-face or phone interviews after discharge.

Covariate data was extracted to analyze for enable adjustment for potential confounders and to define subgroups, including age; sex; health coverage (public, semi-public, or private); dates of symptoms onset, worsening, or hospitalization; pregnancy; immunocompromising conditions; autoimmune or inflammatory disorders; and number and type of comorbidities (Supplementary Sections 3 and 4).

Respiratory swab samples were collected at admission for patients with equal or less than 10 days after the onset of symptoms. Swabs were preserved in a viral transport medium (Universal Transport Medium™, Copan Diagnostics) at 4 °C for up to 24 hours and stored at −70 °C until processing. Samples were tested for SARS-CoV-2 identification by real-time RT-PCR (Kit Molecular SARS-CoV2, Bio-Manguinhos, Rio de Janeiro, Brazil) or by RAT, following standardized procedures. Positive samples were shipped to *Laboratório de Vírus Respiratórios, Exantemáticos, Enterovírus e Emergências Virais* (LVRE)/Fiocruz, a World Health Organization (WHO) reference laboratory for SARS-CoV-2. Positive samples with an elevated viral load, as determined by a real-time RT-PCR cycling threshold below 30, were processed using the following protocols. SARS-CoV-2 genome sequences were generated using the Illumina COVIDSeq Test kit (Illumina, Inc., San Diego, USA) .^13^ Raw data were converted to FASTQ files at Illumina BaseSpace cloud, and consensus sequences were produced with ViralFlow 1.0.^14^ All genomes were evaluated for mutation calling and quality with the Nextclade 2.14 algorithm.^15^ Whole-genome consensus sequences were classified using the “Phylogenetic Assignment of Named Global Outbreak Lineages” (PANGOLIN) software v4.2.^16^

### Exposure

The authors defined the participants who did not receive a dose of any COVID-19 vaccine as unvaccinated, and partially vaccinated as those who received one dose of any COVID-19 vaccine (except for Ad26.COV2.S), ≥ 22 days prior to COVID-19 symptoms onset. Fully vaccinated participants were those vaccinated with a complete primary series according to the schedule approved for each COVID-19 vaccine type and/or boosted with one or two doses under a homologous or heterologous scheme, ≥ 15 days prior to COVID-19 symptom onset.

Homologous schemes comprised a complete primary series and/or booster doses from the same type of COVID-19 vaccine, whereas heterologous schemes included two doses of different COVID-19 vaccines or a complete primary series and a booster from different types of COVID-19 vaccines. Patients with unknown vaccination status or not matching the defined exposure categories were excluded (Supplementary Section 5).

### Outcomes

The primary outcome for LIVE was the detection of SARS-CoV-2 in patients hospitalized with COVID-19-like symptoms, including acute onset of cough, fever, or shortness of breath or anosmia; ageusia or dysgeusia; or general weakness/fatigue, headache, myalgia, sore throat, coryza, dyspnea, anorexia/nausea/vomiting, diarrhea, and altered mental status, according to WHO definition. Hospitalization was defined as admission to the hospital with at least an overnight stay. The secondary outcome was the identification of SARS-CoV-2 genetic variants among positive cases, while the exploratory outcomes were the levels of disease severity according to the WHO Clinical Progression Scale.^17^ Moderate disease was defined as hospitalization without Intensive Care Unit (ICU) admission, oxygen therapy, oxygen requirement by mask or nasal prongs, or in-hospital death. Severe disease included hospitalization cases with ICU admission and without in-hospital death, need for non-invasive and invasive mechanical ventilation, need for hemodynamic support, or need for hemodialysis (Supplementary Section 6).

Readmissions related to continuing symptoms from the original SARS-Cov-2 infection were recorded during the follow-up period. The participants could discontinue the observational study at any time (Supplementary Section 7).

## Results

### Study population characteristics

From February to December 2022, the authors recruited 792 individuals. The authors excluded five patients who were not hospitalized for COVID–19–related reasons and one patient previously hospitalized for COVID-19 within three months of current admission. Study sites in Mexico recruited the most overall participants (60.9 %) and COVID-19 cases (73.5 %). Brazil, Colombia, and Mexico identified between 30.4 % and 34.0 % of the controls (Table 1 and Supplementary Table 1). Across the five countries, 786 individuals were included in the study, 536 COVID-19 cases, and 250 controls. 51 participants were unavailable to contact for follow-up after hospital discharge.

**Table 1:** Demographics and baseline characteristics.

	COVID-19 cases, n ( %)	Controls, n ( %)	Overall, n ( %)
**n participants**	536	250	786
Male	271 (50.6)	113 (45.2)	384 (48.9)
Female	265 (49.4)	137 (54.8)	402 (51.1)
**Testing**			
RT-PCR	177 (33.0)	146 (58.4)	323 (41.1)
Antigen	359 (67.0)	104 (41.6)	463 (58.9)
**Country**			
Mexico	394 (73.5)	85 (34.0)	479 (60.9)
Colombia	85 (15.9)	81 (32.4)	166 (21.1)
Brazil	43 (8.0)	76 (30.4)	119 (15.1)
Panama	8 (1.5)	7 (2.8)	15 (1.9)
Costa Rica	6 (1.1)	1 (0.4)	7 (0.9)
**Vaccination status**			
Unvaccinated	302 (56.3)	95 (38.2)	397 (50.5)
Partially vaccinated	15 (2.8)	13 (5.2)	28 (3.6)
Completed primary series	106 (19.8)	74 (29.6)	180 (22.9)
First booster	85 (15.9)	52 (20.8)	137 (17.4)
Second booster	27 (5.0)	15 (6.0)	42 (5.3)
Missing	1 (0.2)	1 (0.4)	2 (0.3)
**Age at hospital admission (years)**			
18–49	117 (21.8)	107 (42.8)	224 (28.5)
50–64	130 (24.3)	60 (24.0)	190 (24.2)
65–79	158 (29.5)	51 (20.4)	209 (26.6)
80+	131 (24.4)	32 (12.8)	163 (20.7)
**BMI ‒ mean (SD) (*n*=733)**	26.7 (6.2)	26.4 (6.8)	26.6 (6.4)
< 18.5	20 (3.7)	18 (7.2)	38 (4.8)
18.5–25	191 (35.6)	97 (38.8)	288 (36.6)
25–30	169 (31.5)	73 (29.2)	242 (30.8)
≥ 30	107 (20.0)	58 (23.2)	165 (21.0)
**Number of comorbidities**			
0	124 (23.1)	88 (35.2)	212 (27)
1	166 (31.0)	74 (29.6)	240 (30.5)
2	135 (25.2)	45 (18.0)	180 (22.9)
≥ 3	111 (20.7)	43 (17.2)	154 (19.6)
a**Disease severity**			
Moderate	274 (51.1)	174 (69.6)	448 (57.0)
Severe	156 (29.1)	76 (30.4)	232 (29.5)
In-hospital death	106 (19.8)	0 (0.0)	106 (13.5)
**Health coverage**			
Public	461 (86.0)	148 (59.2)	609 (77.5)
Semi-public	4 (0.7)	6 (2.4)	10 (1.3)
Private	68 (12.7)	95 (38.0)	163 (20.7)

RT-PCR, Real-time Polymerase Chain Reaction; BMI, Body Mass Index; SD, Standard Deviation.

a Categories are mutually exclusive.

Females accounted for 51.1 % (402) of the total sample, 49.4 % (265) of cases, and 54.8 % (137) of controls. The mean age at hospital admission was 60.5 (Standard Deviation [SD = 20.1]) years overall, 63.7 (SD = 19.1) years for COVID-19 cases, and 53.8 (SD = 20.4) years for controls; the mean age also varied among countries from 53.8 years in Panama to 65.9 years in Mexico (Supplementary Fig. 1). Overall, 26.6 % and 20.7 % of participants were aged 65–79 and > 80 years, respectively, while the highest number of COVID-19 cases was observed in the 65–79 age group (29.5 %) (Table 1).

### Comorbidities and other risk factors

Data related to the participants’ health profiles and risk factors were collected. Most participants (92.5 %) reported residing in urban areas; of these, 92.7 % were COVID-19 cases and 92.0 % were controlled. Only a small proportion (1.3 %) of all study participants were long-term care facility residents.

**Fig. 2:**
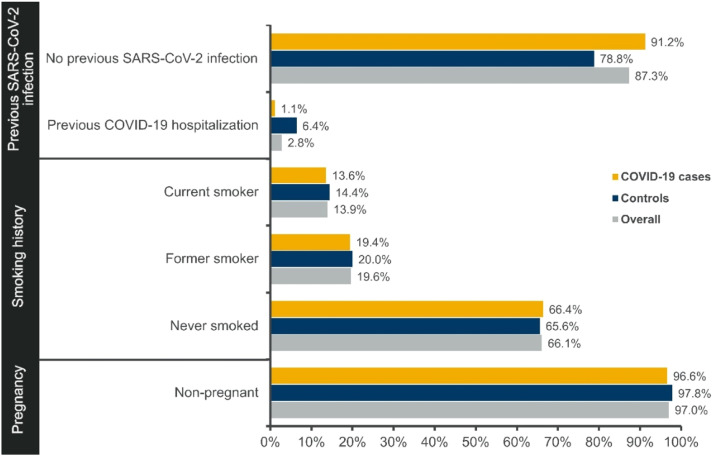
Risk factors.

The overall nutritional status of the study participants was pre-obesity (mean Body Mass Index [BMI = 26.6]; SD = 6.4) according to WHO guidelines. However, the BMI category 18.5–25 (normal weight) was the most frequent when considering COVID-19 cases and controls individually (Table 1). Pregnancy and HIV positivity were reported in only 1.9 % and 1.1 % of all study participants, respectively. Cases and controls reported similar smoking history; among overall participants, 66.1 % had never smoked, 19.6 % were former smokers, and 13.9 % were current smokers (Fig. 2).

Regarding the history of prior SARS-CoV-2 infection, only 2.2 % of overall participants reported infections diagnosed clinically (1.1 % in COVID-19 cases and 4.4 % in controls), and 3.7 % had infections confirmed both clinically and through laboratory testing (2.2 % in COVID-19 cases and 6.8 % in controls). Furthermore, only 2.8 % of the overall participants reported a previous COVID–19–related hospitalization within the past 12 months, with some variation observed between cases (1.1 %) and controls (6.4 %). The majority (97.2 %) of the overall participants, including a slightly higher percentage of COVID-19 cases (98.9 %) compared to controls (93.6 %), did not require hospitalization during their prior infections (Fig. 2).

**Fig. 3:**
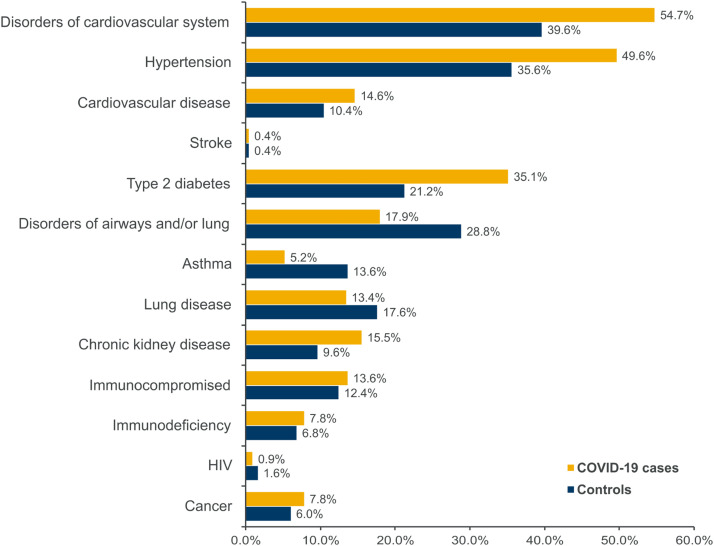
Comorbidities among study participants. Disorders of the cardiovascular system: hypertension, cardiovascular disease, and stroke; Disorders of airways and/or lung: asthma and lung disease; Immunocompromised: immunodeficiency, HIV, and cancer.

Overall, 27.0 % of participants did not report comorbidities, 30.5 % had one, 22.9 % had two, and 19.6 % had three or more comorbidities. The proportion of participants without comorbidities was higher in controls (35.2 %) than in cases (23.1 %) (Table 1). The most common comorbidities were disorders of the cardiovascular system (COVID-19 cases, *n* = 293; controls, *n* = 99), comprising hypertension, cardiovascular disease, and stroke; and type II diabetes (COVID-19 cases, *n* = 188; controls, *n* = 53) (Fig. 3).

### Disease severity and outcomes

The length of hospital stay was evaluated in the COVID-19 group; case participants reported a mean stay of 13.60 (SD = 12.26) days. By disease severity, the highest proportion of COVID-19 cases was hospitalized 10–29 days (27.2 %) for moderate disease, followed by < 10 days for moderate (19.6 %) and severe (14.2 %) disease (Table 2). For hospital stays lasting 10–29 days, the percentage of unvaccinated participants (30.8 %) was higher than those vaccinated (17.4 %), but no substantial differences were observed for the other lengths of hospital stay.

**Table 2:** Hospital length of stay by disease severity.

Length of hospital stay (days)	Disease severity	COVID-19 cases, n ( %)
< 10	Moderate	105 (19.6)
	Severe	76 (14.2)
	Death	46 (8.6)
10–29	Moderate	146 (27.2)
	Severe	58 (10.8)
	Death	54 (10.2)
30–59	Moderate	13 (2.4)
	Severe	19 (3.5)
	Death	6 (1.1)
≥ 60	Moderate	10 (1.9)
	Severe	3 (0.6)
	Death	0 (0.0)
Total		**536 (100)**

All documented deaths occurred within the COVID-19 group; 74.1 % (*n* = 106) were attributed to SARS-CoV-2 infection, while 25.9 % (*n* = 37) were caused by factors other than COVID-19. Mexico reported 96.2 % of the COVID–19–related deaths (*n* = 102), whereas deaths were not reported in Costa Rica and Panama. 122 deaths occurred before discharge and 21 during follow-up. In the COVID-19 group, 51.1 % of cases were moderate (*n* = 274), 29.1 % (*n* = 156) severe, and 19.8 % died (*n* = 106) during hospitalization (Table 1).

Of the 342 cases observed during the follow-up period, 12.0 % (41) were readmitted for continuing symptoms from the original admission with a SARS-CoV-2 infection, and 56.1 % of the patients readmitted were males. The 65–79 age group had the highest percentage of hospital readmissions (43.9 %), followed by the 18–49 age group (22.0 %).

### Vaccination status

In this study, 397 participants (50.5 %) were unvaccinated, 28 (3.6 %) were partially vaccinated, 180 (22.9 %) had completed the primary vaccination schedule (first and second dose), 137 (17.4 %) had received one and 42 (5.3 %) two booster doses. Most COVID-19 cases (56.3 %) were unvaccinated individuals compared to 38.2 % of the controls (Table 1).

**Fig. 4:**
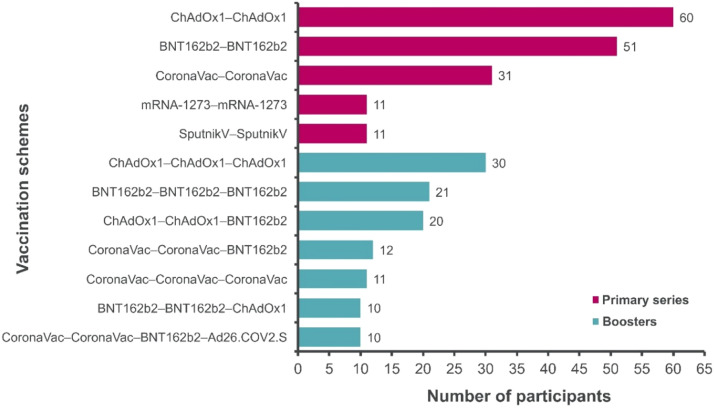
Vaccination schemes by manufacturer: BNT162b2, Pfizer-BioNTech; CoronaVac, Sinovac; mRNA-1273, Moderna; SputnikV, Gamaleya; ChAdOx1, Oxford/AstraZeneca; Ad26.COV2.S, Janssen. Vaccines were administered in order from left to right.

Of the 786 study participants, 15.8 % (124) had received at least one dose of the ChAdOx1 vaccine either in a homologous or heterologous scheme. The most reported primary series were ChAdOx1 (*n* = 60) and BNT162b2 (*n* = 51) homologous schemes. Similarly, among boosted participants, a third dose of ChAdOx1 in a homologous scheme was the most reported (*n* = 30), followed by BNT162b2 homologous booster (*n* = 21) and a heterologous scheme of a ChAdOx1 primary series and a BNT162b2 booster (*n* = 20) (Fig. 4).

Only 6.7 % of overall participants had received their last dose of COVID-19 vaccine ≤ 8 weeks before the onset of the symptoms, and most had surpassed 32 weeks since receiving their last dose in both cases and controls (57.7 % and 57.4 %, respectively) (Table 3).

**Table 3:** Time since the last COVID-19 vaccine dose.

Time (weeks)	COVID-19 cases, n ( %)	Controls, n ( %)	Overall, n ( %)
≤ 8	17 (7.3)	9 (5.8)	26 (6.7)
9–16	18 (7.7)	15 (9.7)	33 (8.5)
17–24	36 (15.4)	20 (12.9)	56 (14.4)
25–32	28 (12.0)	22 (14.2)	50 (12.9)
> 32	135 (57.7)	89 (57.4)	224 (57.6)
**Total**	**234 (100)**	**155 (100)**	**389 (100)**

### Genetic variants and lineage

278 samples of the COVID-19 cases were successfully sequenced; 244 were defined as Omicron, 13 as recombinant variants, and in 21 cases, the variant was not identified due to the limited viral load or incomplete genome sequencing. The most prevalent Omicron lineage was BQ.1.1 (11.9 %), followed by BQ.1.14 (8.6 %), BA.5.1 (6.1 %), BQ.1 (6.1 %), and BA.4.1 (5.4 %). XBB.1.15 was the most prevalent recombinant lineage, accounting for 2.5 % of the positive samples. Other recombinant lineages, namely XBB.1, XAM, XAS, XBB.2.6, and XBB.8, represented smaller proportions, ranging from 0.7 % to 0.4 %. All Omicron and recombinant lineages identified are listed in Supplementary Table 2.

## Discussion

The authors summarized the demographic characteristics of patients admitted with COVID-19-like symptoms in five Latin American countries. Participants in the COVID-19 group were older and more frequently unvaccinated than the controls. Individuals without comorbidities were found at a higher percentage in controls (35.2 %) than in cases (23.1 %). Of the total 786 participants recruited, 73.0 % reported at least one comorbidity.

The main reported comorbidities were disorders of the cardiovascular system, including hypertension, cardiovascular disease, and stroke, followed by diabetes, which are among the most frequent noncommunicable diseases in Latin America^18^ and have been previously identified as major comorbidities in COVID-19 patients from South America.^19^ Throughout the pandemic, hypertension and diabetes have been the principal comorbidities associated with COVID–19–related deaths in Mexico. However, the frequency of other comorbidities changed after vaccine introduction; obesity decreased, whereas cardiovascular disease, chronic kidney disease, chronic obstructive pulmonary disease, and immunosuppression increased among COVID–19–related deaths.^20^

The authors recorded 106 COVID–19–related deaths among the 536 COVID-19 cases recruited, which occurred mostly in Mexico. COVID-19 mortality decreased in this country as the pandemic unfolded and was higher in individuals with comorbidity than in those without comorbidity.^20^ The analysis of a national retrospective cohort reported a reduction of 74.0 %–85.0 % in COVID-19 hospitalizations, deaths, and excess deaths by the fourth wave (Omicron BA.1, BA.2).^20^ As primary-series vaccination and boosters became accessible to the general adult population in Mexico, the mean age of COVID-19 deaths increased. Domínguez-Ramírez et al.^20^ suggested that boosters are more effective in preventing severe COVID-19 in younger than older adults with comorbidity.^20^ In accordance with these observations, the authors estimated that COVID-19 cases were older than controls at hospital admission (63.7 vs. 53.8 years); older adults (65–79 years-old) presented the highest percentage of cases (29.5 %) and readmissions (43.9 %). In addition, moderate disease was more frequent among COVID-19 cases (51.1 %) than severe outcomes (29.1 % severe disease and 19.8 % in-hospital death). The length of hospital stay was similar between vaccinated and unvaccinated participants, except for hospital stays lasting 10–29 days, for which there were more unvaccinated than vaccinated (30.8 % vs. 17.4 %) participants.

Vaccination is not only beneficial for older adults but also for the younger population; however, COVID-19 vaccination programs have stalled globally and as of November 2023, only 67 % of the population received a complete primary series. In Costa Rica, Brazil, Panama, and Colombia, the total population fully vaccinated was 86.0 %, 81.0 %, 74.0 %, and 73.0 %, respectively, while Mexico only reached 63.0 %. The percentage of the total population boosted with at least one dose remains low ranging from 29.0 % in Colombia to 59.0 % in Costa Rica.^21^

Patient recruitment for the LIVE study occurred simultaneously with booster vaccinations in the participating countries. Booster doses of COVID-19 vaccines were available in Brazil and Panama, for elderly adults, HCW, and immunosuppressed patients as of September 2021 and for everyone older than 18 years starting November 202,1.^22^^,^^23^ In Colombia, booster doses were applied from November 2021, prioritizing HCW and other occupations such as professors, personnel of some government departments, police officers, and caregivers of elderly adults, as well as 16–59 year-old individuals with comorbidities, being available to the adult population as of February 202,2.^24^ In Mexico and Costa Rica, booster doses were available from December 2021, first aimed at HCW and elderly people, and gradually distributed to all adults during the first half of 202,2.^25^^,^^26^

The Omicron variant was first identified in November 2021 and rapidly spread worldwide.^1^ The Omicron BA.1 variant was first identified in Latin America in December 2021 and was progressively replaced with BA.2 (weeks 12 to 24, 2022), a combination of BA.4 and BA.5 (weeks 25 to 34, 2022), and further BA.5 sublineages after week 34.^27^ BA.1, BA.2, BA.5, and BQ.1. were the most frequently identified variants in Brazil, Colombia, Costa Rica, Mexico, and Panama, between December 2021 and January 202,3.^11^

The authors sequenced 456 samples, but only 278 were positive; 109 were negative and 69 were not defined, due to the limitations on the viral load or completion of the genome sequence. The most prevalent Omicron variants identified were mainly sublineages of BA.5 (BQ.1.1, BQ.1.14, and BA.5.1); which were common in the region from May 2022 to April 2023.^11^ During the study period, recombinant variants were rarely detected during the surveillance of genetic variants in the participating countries except for Panama, where these variants were abundant around May and June 202,2.^11^ The authors identified XBB.1.15 as the most prevalent recombinant sublineage but only accounted for 2.5 % of the positive samples sequenced.

The emergence of new SARS-CoV-2 variants and the waning immunity among the population have resulted in lower effectiveness of the available vaccines. From December 2021 to September 2022, several Latin American countries reported reduced VE against hospitalization (Chile, Costa Rica, Ecuador, Guatemala, Paraguay, and Uruguay). The adjusted VE of the primary series ranged between 30.0 %–39.6 % for mRNA vaccines and 24.8 %–32.5 % for viral vector vaccines in the adult population. For patients with > 180 days since the last vaccination dose, adjusted VE ranged from −13.8 % to 58.4 % for viral vector vaccines and 3.4 % to 24.4 % for mRNA vaccines.^9^ In the present study, approximately 57.0 % of the participants reported > 32-weeks since their last vaccine dose, and therefore the authors can presume that their immunity declined substantially.

The Omicron variant has shown increased transmissibility and immune escape compared to previous variants.^28^^,^^29^ Moreover, meta-analyses have estimated higher reinfection rates during the Omicron waves compared to other variants, but these rates are only around 3.31 %–4.4 %.^30^^,^^31^ In this study, most participants had comorbidities, and many were unvaccinated, which are risk factors for reinfection;[31] However, only 1.1 %–2.2 % of the cases reported a previous COVID-19 diagnosis. Vaccination remains an effective strategy to protect the population as it has been reported to reduce both the risk of reinfection and severe COVID-19 outcomes.^30^

A major strength of this study is the prospective design, allowing us to concentrate on recent demographic and vaccination data, health profiles, disease outcomes, and SARS-CoV-2 variants of patients hospitalized in Latin America. In addition, the inclusion of a comprehensive regional description of COVID-19 patients hospitalized in Brazil, Colombia, Costa Rica, Mexico, and Panama, rather than focusing on a single country, increases the study's generalizability.

However, there are also limitations. First, sample size estimations to assess VE are challenging as they strongly depend on the SARS-CoV-2 attack rate and the vaccination coverage of COVID-19 vaccines, with both parameters being difficult to predict. As immunity grew in the population due to vaccination and natural infection, COVID-19 cases declined in severity,^28^^,^^29^ which influenced patient recruitment in this study. Compared to Delta, Omicron has shown a lower risk of hospitalization, ICU admission, oxygen therapy, non-invasive and invasive ventilation, and death.^32^ All participating countries reported low rates of COVID-19 cases during the September 2022 monitoring visits. Particularly in Panama, a COVID-19 unit was closed due to the low number of hospitalized patients and withdrawn from the study in August 2022.

Second, variations between the observation period defined in this protocol and that of the study sites reduced the eligible hospitalization episodes. Moreover, some eligible patients chose to be transferred to public hospitals due to the hospitalization costs in private hospitals or sought medical care only when they were in advanced critical condition and declined to participate.

Third, there is potential for bias in the present study as 58.9 % of the participants were tested with RATs, which are generally less sensitive than RT-PCR tests. Among participants tested with RATs, 104 were negative for SARS-CoV-2, representing 13 % of all participants. However, RAT accuracy increases when conducted in symptomatic individuals within the first week of symptom onset.^33^ The present study population included patients hospitalized with COVID-19-like symptoms, and the respiratory swab samples were collected within a maximum of 10 days after the onset of symptoms, limiting bias due to the potential misclassification of outcomes.

## Conclusion

As the authors enter an endemic phase, updated data on risk groups, VE, and duration of protection will be essential to design effective National Immunization Programs. This study collected recent data on the demographic characteristics of COVID-19 patients hospitalized in Latin America. The authors observed that COVID-19 patients were older, with comorbidities, and frequently unvaccinated. Moreover, COVID-19 patients and controls reported > 32 weeks since their last COVID-19 vaccine dose. These data may be useful in establishing target populations and developing public health measures, such as updated vaccines or booster recommendations, robust surveillance, and prompt responses to potential shifts in disease trends.

## Conflicts of interest

BEF, LFTG, LRG, and WM are employees of AstraZeneca and may hold AstraZeneca stock. JVHV, ZAT, PMR, and LES are employees of P95 and have no conflicts of interest to declare. CLM, ELM, MBA, and ARM have no conflicts of interest to declare.

## Appendix Supplementary materials

Image, application 1

## References

[1] LaRotta J., Escobar O., Ávila-Aguero M.L., Torres J.P., R Sini de Almeida, GdC Morales (2023). COVID-19 in Latin America: a snapshot in time and the road ahead. Infect Dis Ther.

[2] Schwalb A., Armyra E., Méndez-Aranda M., Ugarte-Gil C. (2022). COVID-19 in Latin America and the Caribbean: two years of the pandemic. J Intern Med.

[3] Spinardi J., Dantas A.C., Carballo C., Thakkar K., Akoury N.A., Kyaw M.H. (2023). Narrative review of the evolution of COVID-19 vaccination recommendations in countries in Latin America. Africa and the Middle East, and Asia. Infect Dis Ther..

[4] Pan American Health Organization. COVID-19 vaccination in the Americas. 2023. Accessed May 3, 2023. Available from: https://ais.paho.org/imm/IM_DosisAdmin-Vacunacion.asp.

[5] Higdon M.M., Wahl B., Jones C.B., Rosen J.G., Truelove S.A., Baidya A. (2022). A systematic review of coronavirus disease 2019 vaccine efficacy and effectiveness against severe acute Respiratory syndrome coronavirus 2 infection and disease. Open Forum Infect Dis.

[6] Cromer D., Steain M., Reynaldi A., Schlub T.E., Khan S.R., Sasson S.C. (2023). Predicting vaccine effectiveness against severe COVID-19 over time and against variants: a meta-analysis. Nat Commun.

[7] Firouzabadi N., Ghasemiyeh P., Moradishooli F., Mohammadi-Samani S. (2023). Update on the effectiveness of COVID-19 vaccines on different variants of SARS-CoV-2. Int Immunopharmacol.

[8] Nasreen S., Chung H., He S., Brown K.A., Gubbay J.B., Buchan S.A. (2022). Effectiveness of COVID-19 vaccines against symptomatic SARS-CoV-2 infection and severe outcomes with variants of concern in Ontario. Nat Microbiol.

[9] Nogareda F., Regan A.K., Couto P., Fowlkes A.L., Gharpure R., Loayza S. (2023). Effectiveness of COVID-19 vaccines against hospitalisation in Latin America during three pandemic waves, 2021–2022: a test-negative case-control design. Lancet Reg Health Am.

[10] World Health Organization. WHO coronavirus (COVID-19) dashboard. 2023. Accessed May 3, 2023. Available from: https://covid19.who.int/.

[11] Hodcroft E.B. CoVariants: sARS-CoV-2 mutations and variants of interest. 2021. Accessed May 4, 2023. Available from: https://covariants.org/.

[12] Solante R., Alvarez-Moreno C., Burhan E., Chariyalertsak S., Chiu N.-C., Chuenkitmongkol S. (2023). Expert review of global real-world data on COVID-19 vaccine booster effectiveness and safety during the omicron-dominant phase of the pandemic. Expert Rev Vaccin.

[13] Resende P.C., Delatorre E., Gräf T., Mir D., Motta F.C., Appolinario L.R. (2021). Evolutionary dynamics and dissemination pattern of the SARS-CoV-2 lineage B.1.1.33 during the early pandemic phase in Brazil. Front Microbiol.

[14] Dezordi F.Z., Neto A.M., Campos T.D., Jeronimo P.M., Aksenen C.F., Almeida S.P. (2022). ViralFlow: a versatile automated workflow for SARS-CoV-2 genome assembly, lineage assignment, mutations and intrahost variant detection. Viruses.

[15] Aksamentov I., Roemer C., Hodcroft E.B., Neher R.A. (2021). Nextclade: clade assignment, mutation calling and quality control for viral genomes. JOSS.

[16] O'Toole Á., Scher E., Underwood A., Jackson B., Hill V., McCrone J.T. (2021). Assignment of epidemiological lineages in an emerging pandemic using the pangolin tool. Virus Evol.

[17] Marshall J.C., Murthy S., Diaz J., Adhikari N.K., Angus D.C., Arabi Y.M. (2020). A minimal common outcome measure set for COVID-19 clinical research. Lancet Infect Dis.

[18] Perez-Cuevas R., Doubova S.V. (2022). Syndemic nature of COVID-19 in Latin American and Caribbean countries: the challenge ahead. Arch Med Res.

[19] Chenchula S., Vidyasagar K., Pathan S., Sharma S., Chavan M.R., Bhagavathula A.S. (2023). Global prevalence and effect of comorbidities and smoking status on severity and mortality of COVID-19 in association with age and gender: a systematic review, meta-analysis and meta-regression. Sci Rep.

[20] Domínguez-Ramírez L., Sosa-Jurado F., Díaz-Sampayo G., Solis-Tejeda I., Rodríguez-Pérez F., Pelayo R. (2023). Age and comorbidities as risk factors for severe COVID-19 in Mexico, before, during and after massive vaccination. Vaccines.

[21] World Health Organization (2024). https://data.who.int/dashboards/covid19/vaccines?n=c.

[22] Brazil Ministry of Health. Nota técnica n° 27/2021-SECOVID/GAB/SECOVID/MS. 2021. Accessed 11 January 2024. Available from: https://www.gov.br/saude/pt-br/coronavirus/vacinas/plano-nacional-de-operacionalizacao-da-vacina-contra-a-covid-19/notas-tecnicas/2021/nota-tecnica-no-27-2021-secovid-gab-secovid-ms.pdf/view.

[23] Ministry of Health of the Republic of Panama. A buen ritmo avanza aplicación de refuerzo de vacuna contra el Covid-19. 2021. Accessed 11 January 2024. Available from: https://www.minsa.gob.pa/noticia/buen-ritmo-avanza-aplicacion-de-refuerzo-de-vacuna-contra-el-covid-19.

[24] Ministry of Health and Social Protection of Colombia. Plan Nacional de Vacunación contra el COVID-19. 2021. Accessed 11 January 2024. Available from: https://www.minsalud.gov.co/salud/publica/Vacunacion/Paginas/Vacunacion-covid-19.aspx.

[25] Ministry of Health of Mexico. 551. Inicia plan de refuerzo de vacunación contra COVID-19 para personas de 60 años y más. 2021. Accessed 11 January 2024. Available from: https://www.gob.mx/salud/prensa/551-inicia-plan-de-refuerzo-de-vacunacion-contra-covid-19-para-personas-de-60-anos-y-mas.

[26] Ministry of Health of Costa Rica. Terceras dosis COVID-19 son para personal de primera respuesta y hogares de larga estancia, resto de población recibe tercera dosis solo como plan de contingencia. 2021. Accessed 11 January 2024. Available from: https://www.ministeriodesalud.go.cr/index.php/prensa/43-noticias-2021/1175-terceras-dosis-covid-19-son-para-personal-de-primera-respuesta-y-hogares-de-larga-estancia-resto-de-poblacion-recibe-tercera-dosis-solo-como-plan-de-contingencia.

[27] Pan American Health Organization. PAHO Biweekly COVID-19 epidemiological update ‒ 19 April 2023. 2023. Accessed 12 January 2024. Available from: https://www.paho.org/en/documents/paho-biweekly-covid-19-epidemiological-update-19-april-2023.

[28] Mohsin M., Mahmud S. (2022). Omicron SARS-CoV-2 variant of concern: a review on its transmissibility, immune evasion, reinfection, and severity. Medicine.

[29] Arabi M., Al-Najjar Y., Sharma O., Kamal I., Javed A., Gohil H.S. (2023). Role of previous infection with SARS-CoV-2 in protecting against omicron reinfections and severe complications of COVID-19 compared to pre-omicron variants: a systematic review. BMC Infect Dis.

[30] Flacco M.E., Acuti Martellucci C., Baccolini V., De Vito C., Renzi E., Villari P. (2022). Risk of reinfection and disease after SARS-CoV-2 primary infection: meta-analysis. Eur J Clin Invest.

[31] Nguyen N.N., Nguyen Y.N., Hoang V.T., Million M., Gautret P. (2023). SARS-CoV-2 reinfection and severity of the disease: a systematic review and meta-analysis. Viruses.

[32] Relan P., Motaze N.V., Kothari K., Askie L., Le Polain de Waroux O., Van Kerkhove M.D. (2023). Severity and outcomes of Omicron variant of SARS-CoV-2 compared to Delta variant and severity of Omicron sublineages: a systematic review and metanalysis. BMJ Glob Health.

[33] Dinnes J., Sharma P., Berhane S., van Wyk S.S., Nyaaba N., Domen J. (2022). Rapid, point-of-care antigen tests for diagnosis of SARS-CoV-2 infection. Cochrane Database Syst Rev.

