# Supplementary material for: Clinical characteristics, SARS-CoV-2 variants, and outcomes of adults hospitalized due to COVID-19 in Latin American countries
Source: Clinics (Sao Paulo). 2025 Apr 23;80:100648. doi: 10.1016/j.clinsp.2025.100648 (PMC12051657; doi:10.1016/j.clinsp.2025.100648)
Supplement: Supplementary file 1 [file mmc1.docx]

CLINICS-D-24-00718_Supplementary Materials

**Supplementary Materials**

**A. Supplementary Methods**

**Supplementary Section 1** Study sites/study periods.

| **Country** | **Site** | **First Site Subject In (First V1 ‒ consent date)** | **Last Site Subject In (Last V1 ‒ consent date)** | **Last Site Subject Last Visit (LP-LV2)** | **Close-out Visit Date (Close-out Monitoring Report)** |
| --- | --- | --- | --- | --- | --- |
| Brazil | IDOR | 22 Jul 2022 | 15 Dec 2022 | 15 Dec 2022 | 28 Jul 2023 |
| Colombia | CEIP | 1 Feb 2022 | 27 Dec 2022 | 19 Apr 2023 | 28 Jun 2023 |
|  | FNC | 10 Jun 2022 | 28 Nov 2022 | 01 Mar 2023 | 26 Jun 2023 |
| Costa Rica | ICIMED | 18 May 2022 | 19 Aug 2022 | 19 Nov 2022 | 07 Jul 2023 |
| Mexico | IMSS | 18 Aug 2022 | 31 Dec 2022 | 17 Apr 2023 | 29 Jun 2023 |
|  | Hospital Civil | 29 Jun 2022 | 30 Dec 2022 | 29 Mar 2023 | 30 Jun 2023 |
|  | Hospital General Dr. Agustín O´Horan | 09 May 2022 | 22 Sep 2022 | 07 Nov 2022 | 11 Jul 2023 |
| Panamá | INDICASAT | 17 Jun 2022 | 13 Dec 2022 | 08 Mar 2023 | 30 Jun 2023 |

**Supplementary Section 2** Sample size.

The study design and site selection were guided by sample size estimations, which were based on the following assumptions:

• COVID-19 vaccination has no effect on test-negative symptomatic disease etiologies.

• COVID-19 vaccination coverage in test-negative controls is similar to the overall vaccination coverage in the source population.

• High specificity RT-PCR testing is used to ascertain cases and controls.

• Overall health status is similar in test-positive cases and test-negative controls.

• The parameters used in the sample size estimation are consistent with the intended source population (e.g., age-restrictions etc.)

The required number of cases for crude and adjusted Vaccine Effectiveness (VE) estimates was calculated through a simulation-based sample size calculation. The adjusted sample sizes were estimated by inflating the crude sample size by 20%.

|  | **Fully vaccinated vs. unvaccinated** | | | |
| --- | --- | --- | --- | --- |
|  | **Crude minimum number of COVID-19 cases** | **Adjusted minimum number of COVID-19 cases** | **Number of COVID-19 negative controls** | **Total participants per country** |
| **1 control for every 1 case** | 160 | 192 | 192 | 384 |
| **3 controls for every 4 cases** | 180 | 216 | 162 | 378 |
| **1 control for every 2 cases** | 230 | 276 | 138 | 414 |
| **1 control for every 4 cases** | 370 | 444 | 111 | 555 |

The authors propose a sample size of 444 COVID +VE cases, for each country, or 2,220 COVID +VE cases overall (across the five countries), to estimate a minimum VE of 70% with sufficient precision (95% CI width of ≤50%), under the following scenarios:

• Low control: case ratios of ≥ 0.25 (i.e., one control for up to four cases)

• Overall vaccine coverage of ≥ 40% (40% fully vaccinated, 70% receiving at least one dose, and 30% completely unvaccinated) and AZ-specific proportion of ≥ 30% (in each country)

• Allow for adjusted VE estimates (crude sample size estimates inflated by 20%)

These numbers would also allow the calculation of overall strain-specific adjusted VE, where the prevalence of the circulating strain is at least 10% overall, and ≥ 40% in a specific country. While these numbers are sufficient to estimate a minimum VE of 70%, the 95% CI width may exceed 50%. A descriptive analysis was performed when 444 COVID-19-positive cases (across all five countries) were recruited. In order to assess the assumptions underpinning the sample size calculations for the primary analysis, progression reports describing recruitment rates, test-positivity rate, test-negativity rate, overall vaccination coverage, and ChAdOx1 vaccine proportion were generated every two weeks for the first three months, and then every month thereafter.

The sample size calculations presented above indicate the minimum number of cases required to detect a VE of 70%. Budget allowing, the authors aimed to continue recruitment even after the minimum number of cases was reached, to improve the precision of the VE estimates. The sample size calculations were performed using a simulation-based tool, using the following default parameters: Number of cases ranged from 50 to 1,500 with a step size of 10; anticipated VE was 70%; significance level was 5%; and the power (1–β) was 80%.

**Supplementary Section 3** Data collected from patients’ medical records or interviews.

| **Covariate** | **Description** | **Mandatory** |
| --- | --- | --- |
| Age at hospital admission | Calculated based on date of birth and date of admission | x |
| Socioeconomic group | Low, middle, high | x |
| Vaccination status | Vaccine brand, doses, vaccination dates, lot/batch numbers (if possible) | x |
| Age at first vaccination | Age when patient received its first dose | x |
| Sex | Male, female | x |
| Date of symptom onset and worsening | Date when patient start with COVID-19 like case symptoms | x |
| COVID-19 status | SARS-CoV-2 test results (RT-PCR test or Quick-Test Antigen) | x |
| Chronic conditions | See Supplementary Section 4 for definitions | x |
| • Asthma | Binary | x |
| • Lung disease | Binary | x |
| • Cardiovascular disease | Binary | x |
| • Hypertension | Binary | x |
| • Chronic kidney disease | Binary | x |
| • Type 2 diabetes | Binary | x |
| • Cancer | Binary | x |
| • Immunodeficiency (or organ transplant) | Binary | x |
| Current treatment/medication usage | Descriptive | x |
| HIV | Binary | x |
| Previous COVID-19 hospitalization in the last 12 months | Date of previous hospitalization due confirmed COVID-19 | x |
| Residency (urban/rural) | Binary | x |
| Current treatment/medication usage | Descriptive | x |
| Number of households | Numeric | x |
| Occupation | Descriptive | x |
| Health coverage | Public/semi-public/private | x |
| Pregnancy | Binary | x |
| Trimester | First, second, third | x |
| Body Mass Index (BMI) | Continuous | x |
| Smoking history | Never smoker, former smoker (smoke-free for at least 28-days), current smoker | x |
| Vaccination history influenza | Being vaccinated with at least one influenza vaccine within 12-months prior to COVID-19 like case definition hospital admission | X |
| Vaccination history pneumococcus | Year of vaccination | x |
| Long-term care facility residence | Binary |  |
| Healthcare worker | Binary |  |
| Healthcare worker with direct contact to patients | Binary | x |
| Healthcare worker with direct contact to COVID-19 patients | Binary | x |
| Healthcare worker in long-term care facility | Binary | x |
| Ethnicity^a^ | Nominal | x |
| Socioeconomic variables^a^ | Ordinal |  |
| Activity of Daily Living | Barthel index: a simple measure of independence for basic activities of daily living with lower scores representing more dependence on a 20-point scale | X |
| Precautionary health behavior/vaccine hesitancy^a^ | Participants will be asked about their precautionary health behavior (e.g., wearing face masks, using hand sanitizer, going to public places) and vaccine hesitancy | x |
| Previous SARS-CoV-2 infection | Previous SARS-CoV-2 infection (clinically defined or laboratory confirmed) | x |

^a^ Depending on the country/hospital site, this data might not be available.

**Supplementary Section 4** Chronic conditions.

| **Covariate** | **Definition** |
| --- | --- |
| Asthma | • Any of the following diagnostic codes (ICD-10): J45, J46 |
|  | • Including: predominantly allergic asthma, nonallergic asthma, status asthmaticus, acute severe asthma |
|  | • Excluding: acute severe asthma, chronic asthmatic (obstructive) bronchitis, chronic obstructive asthma, eosinophilic asthma, lung diseases due to external agents |
| Lung disease | **• Any of the following diagnostic codes (ICD-10):** A15-16, A19, A31.0, B33.4, E84.0, J40-44, J47, J60-70, J80-84, J85-86, J90-91, J92.9, J93-94, J95-99 |
|  | **• Including:** Tuberculosis (pulmonary, miliary but not that of other systems), atypical mycobacteria, cystic fibrosis, chronic obstructive pulmonary disease, bronchiectasis and other chronic sequelae of infections, chronic lung diseases due to external agents, interstitial lung diseases, pleural diseases, respiratory failure |
|  | **• Excluding:** acute respiratory infections, lung cancer, diseases of pulmonary circulation, pleural plaques without asbestos, previous uncomplicated pneumothorax |
| Cardiovascular disease | **• Any of the following diagnostic codes (ICD-10):** A52.0, B37.6, I01-02, I05-09, I11.0, I13.0, I13.2, I20-25, I26-28, I30-43, I44- 46, I48, I49.0, I49.5, I50-52, I70-71, Q20-Q28 |
|  | **• Including:** all conditions of heart and large vessels that are chronic or likely to have chronic sequelae. Cardiovascular syphilis, endo-, myo- and pericarditis, rheumatic fever, chronic rheumatic heart diseases, congenital malformations, hypertensive (renal) diseases with heart failure, ischemic heart diseases, diseases of pulmonary circulation, atherosclerosis, cardiomyopathies, most conduction disorders, heart failure, aortic aneurysms and dissection, other heart diseases and their complications |
|  | **• Excluding:** uncomplicated hypertension, previous uncomplicated pulmonary embolism (with no lasting cardiac insufficiency), paroxysmal tachycardias, most cases of premature depolarization |
| Hypertension | • Any of the following diagnostic codes (ICD-10): I10, I11.9, I12, I13.1, I13.9, I15 |
|  | • Including: essential (primary) hypertension, secondary hypertension |
|  | • Excluding: hypertensive heart/renal disease with (congestive) heart failure |
| Chronic kidney disease | **• Any of the following diagnostic codes (ICD-10):** I12-13, M10.30, N00-19, N20.0, N25-27, N28.0, N28.9, Q63.9, Z90.5 |
|  | **• Excluding:** clinically nonsignificant kidney cysts |
| Type 2 diabetes | • Any of the following diagnostic codes (ICD-10): E11 |
|  | • INCLUDING: non-insulin dependent diabetes mellitus (adult-onset, maturity-onset, nonketotic, stable, type II, non-insulin-dependent diabetes of the young) |
| Cancer | • Any of the following diagnostic codes (ICD-10): C00-97, D37-48, Z85, Z92.3, Z92.6 |
|  | • Including: all malignant neoplasms (both solid and hematologic) with potential to metastasize, either in treatment, active follow up, or < 5-years post curative treatment |
|  | •Excluding: benign and in situ neoplasms. Basal cell carcinomas. Any cancer previously treated with curative intent and in complete remission for ≥ 5-years |
| Immunodeficiency (or organ transplant) | • Any of the following diagnostic codes (ICD-10): B20-B24, D80-84, D89, Z94 |
|  | • Including: HIV infections, immunodeficiencies and organ transplants. Or iatrogenic: ≥ 2-week systemic treatment, in the 3-months preceding symptom onset, with any of the following: corticosteroid (≥ 20 mg prednisolone daily or equivalent), ciclosporin, tacrolimus, mycophenolate, methotrexate, azathioprine, TNF-α blockers and other biological or cytostatic drugs with immunosuppressive effect |
|  | • Excluding: disorders of the immune system which do not lead to immunosuppression (e.g., some autoimmune conditions) |

**Supplementary Section 5** Vaccination statuses.

Vaccinated participants were aggregated into the following groups:

|  | **Vaccination status** | **Definition** |
| --- | --- | --- |
| 1 | Overall | Anybody who received at least one dose of COVID-19 vaccine irrespective of the total number of doses of ChAdOx1 or any other brand (Groups 2‒10). |
| 2 | Partially completed primary series | Participants vaccinated with only one dose of COVID-19 vaccine ≥22-days prior to COVID-19 symptom onset and no other dose of any other vaccine. |
| 3 | Completed homologous primary series | Participants vaccinated with two doses of the same COVID-19 vaccine with the most recent dose administered ≥15-days prior to COVID-19 symptom onset. |
| 4 | Completed heterologous primary series | Participants vaccinated with two doses in total of different COVID-19 vaccines and no doses of Ad26.COV2.S. Additionally, the most recent dose was administered ≥15 days prior to COVID-19 symptom onset. |
| 5 | First booster, homologous series (3^rd^ dose) | Participants vaccinated with three doses in total of the same COVID-19 vaccine for who the most recent dose was administered ≥15-days prior to COVID-19 symptom onset. |
| 6 | First booster (ChAdOx1), heterologous series (3^rd^ dose, or 2^nd^ dose if primary series was Ad26.COV2.S) | Participants vaccinated with three doses in total of COVID-19 vaccines including at least two brands and one dose of ChAdOx1 as a booster dose. The only exception to this rule was that participants vaccinated with Ad26.COV2.S as the first dose, ChAdOx1 as the second dose, and no other doses of any other vaccine were also included in this group. The most recent dose had to be administered ≥ 15-days prior to COVID-19 symptom onset. |
| 7 | First booster (non-ChAdOx1), heterologous series (3^rd^ dose, or 2^nd^ dose if primary series was Ad26.COV2.S) | Participants vaccinated with three doses in total of COVID-19 vaccines including at least two brands, and a non-ChAdOx1 booster dose. The most recent dose had to be administered ≥15-days prior to COVID-19 symptom onset. |
| 8 | Second booster, homologous series (4^th^ dose) | Participants vaccinated with four doses in total of the same COVID-19 vaccine, for who the most recent dose was administered ≥15-days prior to COVID-19 symptom onset. |
| 9 | Second booster (ChAdOx1), heterologous series (4^th^ dose, or 3^rd^ dose if primary series was Ad26.COV2.S) | Participants vaccinated with four doses in total of COVID-19 vaccines including at least two brands and an ChAdOx1 dose as the second booster. The only exception to this rule was that participants vaccinated with Ad26.COV2.S as the first dose and three doses in total were also included in this group. The most recent dose was administered ≥ 15-days prior to COVID-19 symptom onset. |
| 10 | Second booster (non- ChAdOx1), heterologous series (4^th^ dose, or 3^rd^ dose if primary series was Ad26.COV2.S) | Participants vaccinated with four doses in total of COVID-19 vaccines including at least two brands and non-ChAdOx1 dose as the second booster. The only exception to this rule was that participants vaccinated with Ad26.COV2.S as the first dose, ChAdOx1 as the second dose, and a non-ChAdOx1 third dose were also included in this group. The most recent dose was administered ≥ 15-days prior to COVID-19 symptom onset. |

**Supplementary Section 6** Primary, secondary, and exploratory outcomes.

| **Outcomes** | **Definition** |
| --- | --- |
| Primary outcomes |  |
| Test-positive case | Participants who met the COVID-19-like case definition and tested positive for at least one SARS-CoV-2 RT-PCR test or Quick-Test (antigen) with specimens collected between 14-days prior to and including within 24 hours of the day of hospital admission (day 0) |
| Test-negative control | Participants who met the COVID-19-like case definition and tested negative for all SARS-CoV-2 RT-PCR or Quick-Test (antigen) tests with specimens collected between 14-days prior to and including a negative test the day of hospital admission (day 0) |
| Secondary outcomes |  |
| Detection of SARS-CoV-2 genetic variants in test-positive cases | |
| Exploratory outcomes | |
| Moderate disease | Hospital admission without ICU admission and without in-hospital death |
|  | Without oxygen therapy |
|  | With oxygen requirement by mask or nasal prongs |
| Severe disease | Hospital admission with ICU admission and without in-hospital death |
|  | Non-invasive and invasive mechanical ventilation |
|  | Need of hemodynamic support |
|  | Need of hemodialysis |
| Dead | A hospitalized participant with in-hospital death |
| Length of hospital stay | The number of overnights spent at the hospital from admission until discharge or death. In case of a referral to another hospital, the date of hospital admission was defined as the date of first admission. COVID-19 hospitalizations within 3-months of the first admission was considered part of the same episode |

**Supplementary Section 7** Study procedures.

| **Variables** | **Visit 1: Day (0) (Hospitalization)** | **Hospital discharge** | **Visit 2: Follow up (3 months after hospital discharge or in case of death)** |
| --- | --- | --- | --- |
| Inclusion/exclusion criteria | X^a,b^ |  |  |
| Inform Consent Form signature | X^a,b^ |  |  |
| COVID-19 PCR for variant evaluation | X^a^ |  |  |
| Date of COVID-19 diagnosis | X^a^ |  |  |
| Previous COVID-19 hospitalization in the last 12 months | X^a,b^ |  |  |
| Vaccination status (partially, fully, or recently) | X^a,b^ |  |  |
| Demographic data (age, sex, residency, BMI, smoking history, ethnicity) | X^a,b^ |  |  |
| Chronic conditions | X^a,b^ |  | X^a^ |
| Current Treatment/medication usage | X^a,b^ |  | X^a^ |
| Activity of Daily Living survey | X^a,b^ |  | X^a^ |
| Precautionary health behavior survey | X^a,b^ |  | X^a^ |
| PROMIS10 Global Health PRO survey | X^a,b^ |  | X^a^ |
| Pregnancy and/or puerperium status | X^a,b^ |  |  |
| ICU admission | X^a^ |  | X^a^ |
| COVID-19 related deaths | X^a^ |  | X^a^ |
| Discharge from hospital date |  | X^a^ | X^a^ |

^a^ For case group should be positive between 14 days prior to and including within 24h of day of hospitalization (day 0).

^b^ For control group should be negative between 14 days prior to and including the day at hospital admission (day 0).

**Voluntary withdrawal/discontinuation**

Patients could be discontinued from this study at any time due to the following reasons:

• Withdrawal of consent by the patient without prejudice to further treatment

• Withdrawal of consent by next of kin/legal representative (for deceased patients at study entry, unless waiver is granted)

• Violation of eligibility criteria

Participants who withdrew from the study were asked about their reasons, which were recorded in the electronic data capture system. If possible, the reasons were assessed by the Investigator according to current practice. The extracted data for the patient, if any, was not used in the analyses for such patients. Discontinued patients were not replaced, and patient management was expected to continue according to clinical practice even if they did not participate in the study.

**Supplementary Section 8** IRB approvals.

The study protocol and informed consent were reviewed and approved by the following institutional review boards:

1. Colombia, Bogotá: “Fundación Neumológica Colombiana.” Located at Cra. 13b #161-85, Bogotá, Cundinamarca. The study was approved by the “Comité de Ética en Investigación Clínica de la Fundación Cardioinfantil, Instituto Cardiologia” on January 28, 2022.

2. Colombia, Cali: “Centro de Estudios en Infectología Pediátrica S.A.S” located at Calle 5b 5 #37 BIS-28, Cali-Valle, Colombia. The study was approved by the “Comité de Ética en Investigación Biomédica de la Corporación Científica Pediátrica” on January 18, 2022.

3. Mexico, Mérida: “Hospital General Dr. Agustín O’Horán” located at Avenida Itzaes s/n x Av Centro Jacinto Canek, C.P 97000 Mérida, Yucatán., México. The study was approved by COFEPRIS and its committees involved as follows: a) “Comité de Ética en Investigación (CEI)” on March 22, 2022, b) “Comité de Investigación (CI)” on March 22, 2022, and c) “Comité de Bioseguridad” on March 17, 2022.

4. Mexico, Guadalajara: “Hospital Civil Fray Antonio Alcalde” located at Hopital #278, Col. Centro. El Retiro, C.P. 44280 Guadalajara, Jalisco, México. The study was approved by COFEPRIS and its committees involved as follows: a) “Comité de Ética en Investigación (CEI)”, b) “Comité de Investigación (CI)” and c) “Comité de Bioseguridad” on February 16, 2022.

5. Mexico, Ciudad de México DF: “UMAE Hospital de Especialidades Centro Médico Nacional Siglo XXI” Av. Cuauhtémoc 330 Col. Doctores, C.P 06720, Cuauhtémoc, Ciudad de México. The study was approved by COFEPRIS and its committees involved as follows: a) “Comité de Ética en Investigación (CEI)”, b) “Comité de Investigación (CI)” and c) “Comité de Bioseguridad” on July 25, 2022.

6. Brazil, São Paulo: “Instituto D’Or de Pesquisa e Ensino” located in Av. República do Líbano, 611 – Ibirapuera, São Paulo – SP, 04501-000. The study was approved by the “Ministério da Saúde – Conselho Nacional de Saúde – Comissão Nacional de Ética em Pesquisa – CONEP” on January 03, 2022.

7. Costa Rica, San José: “Instituto de Investigación en ciencias médicas UCIMED” located at De la Pops de Sabana, 400 metros oeste, San José; 2296-0100. The study was approved by the “Comité Ético Cientifico – CEC – UCIMED” on January 20, 2022.

8. Panamá, Ciudad de Panamá: “INDICASAT” located in Hospital Santo Tomás Calle 34 Este Panamá. Sitio de respaldo Unidad de Investigación Clínica INDICASAT-AIP Corregimiento de Pueblo Nuevo, Urbanización La Loma, Calle 69 oeste casa # 76 Panamá. The study was approved by the “Comité de Bioética de la Investigación del Instituto Conmemorativo GORGAS de Estudios de la Salud (CBI-ICGES)” on April 01, 2022.

**B. Supplementary Tables and Figures**

**Supplementary Table 1** Recruitment by study site.

| **Site** | **Overall** | **COVID-19 cases** | **Controls** |
| --- | --- | --- | --- |
| CEIP | 133 (16.9%) | 64 (11.9%) | 69 (27.6%) |
| Hospital Agustín O’Horán | 27 (3.4%) | 18 (3.4%) | 9 (3.6%) |
| ICIMED | 7 (0.9%) | 6 (1.1%) | 1 (0.4%) |
| FNC | 33 (4.2%) | 21 (3.9%) | 12 (4.8%) |
| Indicasat | 15 (1.9%) | 8 (1.5%) | 7 (2.8%) |
| IDOR | 119 (15.1%) | 43 (8.0%) | 76 (30.4%) |
| Hospital Civil | 54 (6.9%) | 39 (7.3%) | 15 (6.0%) |
| IMSS | 398 (50.6%) | 337 (62.9%) | 61 (24.4%) |
| Total | **786 (100%)** | **536 (100%)** | **250 (100%)** |

**Supplementary Table 2** Variants and lineage for COVID-19 cases.

| **Variant** | **Lineage** | **n (%^a^)** |
| --- | --- | --- |
| Not defined | Not defined | 21 (7.55) |
| Recombinant | XBB.1.15 | 7 (2.52) |
|  | XBB.1 | 2 (0.72) |
|  | XAM | 1 (0.36) |
|  | XAS | 1 (0.36) |
|  | XBB.2.6 | 1 (0.36) |
|  | XBB.8 | 1 (0.36) |
| Omicron | BQ.1.1 | 33 (11.87) |
|  | BQ.1.14 | 24 (8.63) |
|  | BA.5.1 | 17 (6.12) |
|  | BQ.1 | 17 (6.12) |
|  | BA.4.1 | 15 (5.40) |
|  | BW.1 | 9 (3.24) |
|  | BA.5.2.9 | 6 (2.16) |
|  | BA.5.6 | 6 (2.16) |
|  | BU.1 | 6 (2.16) |
|  | BA.5.1.23 | 5 (1.80) |
|  | BA.5.1.6 | 4 (1.44) |
|  | BA.5.2.1 | 4 (1.44) |
|  | BA.5.2.23 | 4 (1.44) |
|  | BN.1 | 4 (1.44) |
|  | BN.1.3.1 | 4 (1.44) |
|  | BQ.1.1.10 | 4 (1.44) |
|  | BW.1.1.1 | 4 (1.44) |
|  | BA.1.1 | 3 (1.08) |
|  | BA.4.1.8 | 3 (1.08) |
|  | BA.5.1.10 | 3 (1.08) |
|  | BE.10 | 3 (1.08) |
|  | BQ.1.1.18 | 3 (1.08) |
|  | BQ.1.2 | 3 (1.08) |
|  | BA.2 | 2 (0.72) |
|  | BA.2.12.1 | 2 (0.72) |
|  | BA.4 | 2 (0.72) |
|  | BA.4.6 | 2 (0.72) |
|  | BA.5.1.25 | 2 (0.72) |
|  | BA.5.2 | 2 (0.72) |
|  | BF.10 | 2 (0.72) |
|  | BQ.1.1.5 | 2 (0.72) |
|  | BQ.1.1.52 | 2 (0.72) |
|  | BQ.1.1.69 | 2 (0.72) |
|  | BQ.1.11 | 2 (0.72) |
|  | BQ.1.5 | 2 (0.72) |
|  | BW.1.1 | 2 (0.72) |
|  | BW.1.1.2 | 2 (0.72) |
|  | DN.2 | 2 (0.72) |
|  | BA.1.15 | 1 (0.36) |
|  | BA.2.1 | 1 (0.36) |
|  | BA.4.2 | 1 (0.36) |
|  | BA.4.4 | 1 (0.36) |
|  | BA.5.1.1 | 1 (0.36) |
|  | BA.5.1.30 | 1 (0.36) |
|  | BA.5.2.22 | 1 (0.36) |
|  | BA.5.2.52 | 1 (0.36) |
|  | BE.1 | 1 (0.36) |
|  | BE.1.4 | 1 (0.36) |
|  | BE.2 | 1 (0.36) |
|  | BF.13 | 1 (0.36) |
|  | BF.5 | 1 (0.36) |
|  | BF.7 | 1 (0.36) |
|  | BF.8 | 1 (0.36) |
|  | BN.1.3.6 | 1 (0.36) |
|  | BN.1.4 | 1 (0.36) |
|  | BQ.1.1.13 | 1 (0.36) |
|  | BQ.1.1.4 | 1 (0.36) |
|  | BQ.1.13.1 | 1 (0.36) |
|  | BQ.1.3 | 1 (0.36) |
|  | CH.1.1.14 | 1 (0.36) |
|  | CK.1 | 1 (0.36) |
|  | CK.2.1.1 | 1 (0.36) |
|  | CM.5.2 | 1 (0.36) |
|  | CQ.2 | 1 (0.36) |
|  | CV.2 | 1 (0.36) |
|  | DL.1 | 1 (0.36) |
|  | EF.1 | 1 (0.36) |
|  | XBB.1.5 | 1 (0.36) |

^a^ Percentage of each variant lineage among 278 samples with a positive sequencing result.

**Supplementary Figure 1** Mean age at hospital admission by country.
